# Symptom burden and splenomegaly in patients with myelofibrosis in the United States: a retrospective medical record review

**DOI:** 10.1002/cam4.136

**Published:** 2013-10-05

**Authors:** Debanjali Mitra, James A Kaye, Lance T Piecoro, Jennifer Brown, Kelly Reith, Tariq I Mughal, Nicholas J Sarlis

**Affiliations:** 1 RTI Health SolutionsResearch Triangle Park, North Carolina; 2 Incyte CorporationWilmington, Delaware; 3 Tufts University School of MedicineBoston, Massachusetts

**Keywords:** Comorbidities, myelofibrosis, myeloproliferative neoplasm, splenomegaly, symptoms

## Abstract

Myelofibrosis (MF) is a clonal hematopoietic malignancy characterized by constitutional and localized symptoms, progressive splenomegaly, bone marrow fibrosis, and cytopenias. Although MF is well studied, few studies exist regarding its symptomatic burden in routine clinical practice. This study aimed to characterize symptoms and other clinical features of MF among patients in the United States. We conducted a retrospective medical record review of adult patients with an MF diagnosis between 1 January 2005 and 31 March 2010, stratified by the presence of palpable splenomegaly. Eligible patients had 12 months or more of follow-up after diagnosis (or after detection of splenomegaly, if present) unless death occurred. Demographic and clinical characteristics, MF-related symptoms, and treatments were reported by treating physicians. We report on 180 MF patients: 102 with splenomegaly, 78 without. Median age was 66 years, 63% were male, and 82% had intermediate-2 or high-risk MF (International Prognostic Scoring System). Fatigue was reported by ∼85% of patients; weight loss, night sweats, and fever (any grade) were each reported by 50% or more of patients. Generalized abdominal pain, left subcostal pain, and early satiety occurred more frequently among patients with splenomegaly. Multiple symptoms were reported by 95% of patients. Common comorbidities were hypertension, diabetes, and chronic pulmonary disease. Symptoms are common in MF patients, regardless of the presence of palpable splenomegaly. Careful assessment of symptom burden is an important aspect of the clinical evaluation of patients with MF.

## Introduction

Myelofibrosis (MF) is a *BCR-ABL1*-negative myeloproliferative neoplasm (MPN) in which clonal proliferation of abnormal hematopoietic stem cells results in progressive bone marrow fibrosis, cytopenias, extramedullary hematopoiesis, and a risk of transformation to acute myeloid leukemia [1]. MF occurs de novo (primary MF [PMF]) or can develop in patients with polycythemia vera or essential thrombocythemia [1, 2]. Patients with MF develop a hypercatabolic state with progressively debilitating symptoms, including constitutional (fever, night sweats, weight loss) and general (fatigue and lethargy) symptoms, and organ-localized complaints [3–5]. Splenomegaly and, to a lesser extent, hepatomegaly are cardinal signs of MF [6]. Symptoms resulting from organ enlargement include abdominal pain, left subcostal pain, abdominal fullness, and early satiety [7], with complications of the disease leading to clinical manifestations of portal hypertension and bleeding from esophageal varices [8].

The estimated incidence of PMF varies from approximately 0.5 per 100,000 in the United Kingdom (1984–1993) [9] to 1.5 per 100,000 in Olmsted County, Minnesota (1976–1995) [10]. Incidence increases markedly with age, from less than 0.1 per 100,000 younger than 45 years to more than 20 times that at ages 75 through 79 years [9]. Median survival has been reported to be approximately 6 years overall, varying from about 2 years for high-risk to 11 years for low-risk disease, according to the International Prognostic Scoring System (IPSS) for PMF [6].

Until recently, there were no regulatory health authority-approved medicines for MF, and the only treatment with a potential to increase survival was allogeneic hematopoietic stem cell transplantation (rarely used because of its high risk in older patient populations). The Janus kinase (JAK) 1 and JAK2 inhibitor ruxolitinib was approved by the United States (U.S.) Food and Drug Administration in November 2011 for newly and previously diagnosed patients with intermediate or high-risk MF (all subtypes), on the basis of the results of two randomized clinical trials in which ruxolitinib reduced splenomegaly, improved MF-related symptoms, and ameliorated several measures of health-related quality of life compared with placebo (COMFORT-I study) or best available therapy (COMFORT-II study) [11, 12]. In both studies, ruxolitinib also was found to improve overall survival versus placebo (COMFORT-I) and best available therapy (COMFORT-II), suggesting an overall survival advantage [13, 14].

To better understand the clinical characteristics and symptom burden of patients with MF who might benefit from new treatments for this disease in real-world settings and to explore the relationship of splenomegaly to patients' symptoms, we undertook a retrospective, observational study of a broad sample of MF patients with or without splenomegaly who were treated in the U.S. before the availability of ruxolitinib.

## Methods

### Study design, data source, and human subjects' protection

Data for this observational cohort study were abstracted by physicians from their patients' medical records. In collaboration with RTI Health Solutions, Kantar Health identified and recruited 57 medical oncologists, hematologists, and hematology–oncology specialists from their *All Global* Panel (including community and academic centers across the U.S.) and collected data electronically. Physicians were required to have been in practice for between 2 and 40 years and to treat at least five patients with MF annually. Each physician abstracted charts for one to eight unique patients (*N* = 216) between April 2011 and May 2011.

All physician and patient data were de-identified; because data were collected retrospectively, study procedures had no influence on prescribing practices or patterns of follow-up care. The study was conducted by RTI Health Solutions, a business unit of RTI International, and the study was determined to be exempt from informed consent requirements by its institutional review board. The study was funded by Incyte Corporation, the manufacturer of ruxolitinib.

### Patient eligibility

Patients were required to have had a diagnosis of PMF, post-polycythemia vera MF (PPV-MF), or post-essential thrombocythemia MF (PET-MF) between 1 January 2005 and 31 March 2010. To obtain sufficient information on symptoms among patients with and without splenomegaly, half of the patients were purposefully selected with splenomegaly. Patients were required to have at least 12 months of medical record information available following the first diagnosis of MF and 12 months or more of records following first evidence of splenomegaly (spleen palpable below the costal margin), if present. However, in order to avoid bias in assessing the frequency of MF symptoms, patients who died during the target minimum period of follow-up were included in the study. Patients who participated in MF-related clinical trials assessing the efficacy or safety of specific treatments during the study follow-up period were excluded.

As is typical in retrospective medical record review studies, the patient sample selected for this study represented a “convenience” sample (selected based on accessibility). However, we endeavored to ensure that all four U.S. Census regions (Northeast, South, Midwest, and West) were well represented by restricting physicians in each region to contributing 15–35% of patients altogether. Although every attempt was made to ensure variation in medical specialty (medical oncologists, hematologists, or hematology–oncology specialists) and geographic region of the physicians contributing data, it cannot be ensured that the selected MF patients were fully representative of the U.S. population.

### Study measures

Baseline information abstracted by study physicians at the time of MF diagnosis included demographic characteristics (age, sex, race, education, employment status, and type of health insurance) and baseline medical history (type of MF [PMF, PPV-MF, or PET-MF]), results of *JAK2* V617F mutation testing, and bone marrow biopsy results. In addition, each patient's MF risk level was computed post hoc at baseline from the IPSS [6]. The IPSS assigns one point each to the following patient characteristics: (a) age older than 65 years, (b) hemoglobin levels under 10 g/dL, (c) white blood cell counts over 25 × 10^9^/L, (d) peripheral blood blasts greater than 1%, and (e) presence of constitutional symptoms (weight loss, night sweats, or fever). Patients then were categorized into risk groups on the basis of the number of adverse prognostic factors: low (IPSS = 0), intermediate-1 (IPSS = 1), intermediate-2 (IPSS = 2), or high (IPSS ≥3). For patients with splenomegaly, the time between MF diagnosis and diagnosis of splenomegaly was documented. Any records of splenectomy, splenic irradiation, or transformation to acute leukemia also were collected. Commonly occurring comorbidities documented at baseline included 17 diagnostic categories comprising the Charlson Comorbidity Index [15]. Symptoms commonly associated with MF were recorded, including principal items from the Myeloproliferative Neoplasm Symptom Assessment Form (MPN-SAF) [16]. Pharmacotherapy for MF and time from diagnosis to treatment initiation were recorded; therapies of interest were hydroxyurea, interferon-α, thalidomide, lenalidomide, anagrelide, and hematopoietic growth factors.

Relevant characteristics of participating physicians were noted, including number of years in practice, medical specialty, current MF case load (by individual physicians and by practice), region of practice, and type of practice (e.g., community clinic, academic hospital).

### Statistical analysis

The study was primarily descriptive and continuous variables were summarized as means, standard deviations, medians, and ranges; categorical variables were reported as frequency distributions. The sample of patients was analyzed overall and separately by splenomegaly status (with vs. without). All analyses were carried out using SAS (version 9.1.3, SAS Institute, Inc., Cary, NC). Differences in symptom prevalence and laboratory values between patients with and without splenomegaly were assessed using chi-square test for categorical variables and *t*-test or Fisher's exact test for continuous variables. This study was not designed to assess causality between the presence of splenomegaly and other parameters of interest.

## Results

### Physician and patient characteristics

Altogether, 216 patient records were abstracted by 57 physicians; 81% of the physicians were hematologists–oncologists; 16%, medical oncologists; and 3%, hematologists. Nearly 65% of the physicians were from community practices; 33% were from academic hospitals. The physicians' practices were distributed among the major U.S. Census regions (South, 33%; Northeast, 29%; West, 21%; Midwest, 18%). Physicians had an average 13.5 years of practice experience; 88% reported treating more than 10 patients with MF in the year before their participation in this study (by design, physicians had to treat at least five MF patients annually). Because of inconsistencies in the reported data, 36 patients could not be unambiguously categorized as having splenomegaly and were consequently excluded from the analysis. Therefore, results were obtained from 180 patients (78 without splenomegaly; 102 with splenomegaly).

Table 1 presents the demographic characteristics of patients included in the analysis: the median age at diagnosis was 66 years and 63% of patients were men. A greater proportion of patients with splenomegaly were white (74%) than those without splenomegaly (58%). There were fewer Hispanic (2%) patients in the group with splenomegaly than in the group without splenomegaly (14%). More than half of all patients were insured by Medicare (57%); the next largest group carried commercial insurance (30%). Approximately 33% of patients were employed, 47% were retired, and 17% were unemployed, with no substantial difference by splenomegaly status. The median follow-up period available from the time of MF diagnosis was nearly 24 months overall. Because of the requirement for additional follow-up time in the splenomegaly group, this was somewhat longer for patients with splenomegaly (28 months) than for those without splenomegaly (20 months).

**Table 1:** Patient characteristics at the time of MF diagnosis

Characteristic (*n*, %)	With splenomegaly (*n*=102)	Without splenomegaly (*n*=78)
Age at diagnosis, median (range)	66 (37–90)	66 (35–89)
<55years	7 (7)	11 (14)
55–64years	38 (37)	21 (27)
65years and older	57 (56)	46 (59)
Sex		
Male	68 (67)	46 (59)
Female	34 (33)	32 (41)
Race/ethnicity		
White	75 (74)	45 (58)
African American	19 (19)	18 (23)
Hispanic	2 (2)	11 (14)
Other	6 (6)	4 (5)
Primary insurance^1^		
Commercial	32 (31)	22 (28)
Medicare	57 (56)	45 (58)
Medicaid	8 (8)	9 (12)

MF, myelofibrosis.

1 Primary insurance for medical benefits. Remaining patients were listed as uninsured, other, or unknown.

### Clinical features and comorbid conditions

Table 2 presents an overview of disease characteristics at the time of MF diagnosis. Patients with PMF constituted 70% of those with splenomegaly and 60% of those without splenomegaly. Overall, 168 (93%) patients were tested for the *JAK2* V617F mutation, and 161 had known results: 106 (66%) were positive and 55 (34%) were negative. There was no material difference in the proportion of *JAK2* V617F-positive patients between the two groups. Among those with splenomegaly, 74% had a palpable spleen length of 10 to 20 cm and 4% of 20 cm or larger. Overall, 87% of patients had a bone marrow biopsy. The median time from MF diagnosis to biopsy was 10 days.

**Table 2:** Disease characteristics at the time of MF diagnosis

Characteristic (*n*, %)	With splenomegaly (*n*=102)	Without splenomegaly (*n*=78)
MF subtype		
PMF	71 (70)	47 (60)
PPV-MF	19 (19)	22 (28)
PET-MF	10 (10)	9 (12)
Unknown	2 (2)	0
IPSS risk category^1^		
Low	4 (4)	4 (5)
Intermediate-1	10 (10)	9 (12)
Intermediate-2	29 (28)	23 (29)
High	56 (55)	39 (50)
Unknown	3 (3)	3 (4)
*JAK2* V617F testing		
Positive	60 (59)	46 (59)
Negative	29 (28)	26 (33)
Not conducted	8 (8)	4 (5)
Unknown	5 (5)	2 (3)
Bone marrow biopsy collected	91 (89)	66 (85)
Fibrosis grade (% of those with biopsy)		
0	0	7 (11)
1	30 (33)	26 (39)
2	49 (54)	26 (39)
3	9 (10)	5 (8)
Unknown	3 (3)	2 (3)

Hg, hemoglobin; IPSS, International Prognostic Scoring System; JAK, Janus kinase; MF, myelofibrosis; PET-MF, post-essential thrombocythemia myelofibrosis; PMF, primary myelofibrosis; PPV-MF, post-polycythemia vera myelofibrosis; WBC, white blood cell.

1 Calculated based on data provided (not physician-reported). IPSS risk categories: one point each assigned to age >65years, Hg <10g/dL, WBC counts >25×10^9^/L, peripheral blood blasts >1%, presence of constitutional symptoms (weight loss, night sweats, fever). Categories defined as: low, IPSS=0; intermediate-1, IPSS=1; intermediate-2, IPSS=2; high, IPSS ≥3.

Four patients in the study sample underwent splenectomy (Table 3); the median time to surgery was ∼6 months after splenomegaly diagnosis. Four patients underwent splenic irradiation. Leukemic transformation was reported to have occurred in four patients (all with splenomegaly) a median of 1.4 years after MF diagnosis.

**Table 3:** Time to diagnosis, treatment, and outcomes associated with splenomegaly in MF patients

Parameter	*n*=102
Time (weeks) between MF diagnosis and detection of splenomegaly, median (range)	0.14 (0.14–119.6)
Splenectomy, *n* (%)	7 (6.9)
Splenic irradiation, *n* (%)	4 (4)
Leukemic transformation, *n* (%)	4 (4)

MF, myelofibrosis.

Table 4 presents the comorbidities noted at the time of MF diagnosis. The most common were hypertension (51%), diabetes (23%), and chronic pulmonary disease (18%), with no substantial difference between the two groups. Nearly 52% of patients had two or more comorbidities.

**Table 4:** Comorbidities at the time of MF diagnosis

Comorbidity (*n*, %)	With splenomegaly (*n*=102)	Without splenomegaly (*n*=78)
Hypertension	53 (52)	39 (50)
Diabetes	21 (21)	20 (26)
Chronic pulmonary disease	20 (20)	13 (17)
Depression	15 (15)	9 (12)
Congestive heart failure	15 (15)	8 (10)
Peripheral vascular disease	8 (8)	8 (10)
Cerebrovascular disease	8 (8)	8 (10)
Myocardial infarction	9 (9)	5 (6)
Ulcer disease	9 (9)	4 (5)
Dementia	4 (4)	5 (6)

MF, myelofibrosis.

### Myelofibrosis-related symptoms

**Figure 1:**
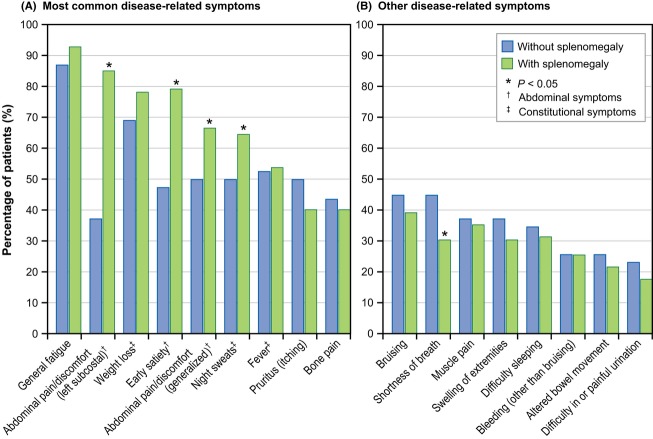
Prevalence of myelofibrosis (MF)-related symptoms at time of diagnosis of splenomegaly or at time of MF diagnosis in patients without splenomegaly. (A) Most common disease-related symptoms, (B) other disease-related symptoms.

**Figure 2:**
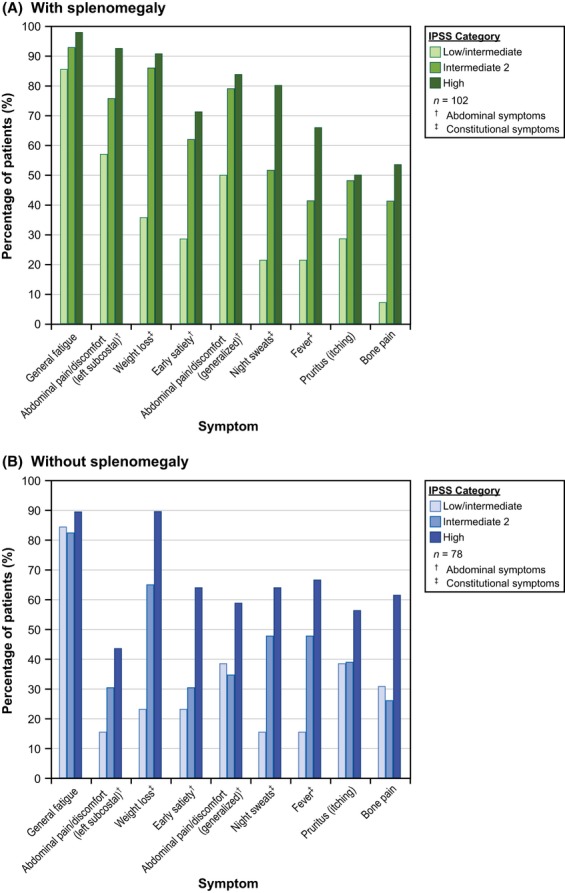
Prevalence of common myelofibrosis (MF)-related symptoms by International Prognostic Scoring System (IPSS) risk category (A) with splenomegaly and (B) without splenomegaly. Symptom prevalence at the time of MF diagnosis and IPSS risk stratification. In patients with splenomegaly, splenomegaly was most often recorded at the time of diagnosis (median time from MF diagnosis to reported splenomegaly was 1 day).

Figure 1 shows the commonly occurring symptoms recorded at the time of splenomegaly detection in patients with splenomegaly and at the time of MF diagnosis in patients without splenomegaly. The most frequently reported symptoms observed in patients with splenomegaly versus those without splenomegaly were generalized fatigue (93% vs. 87%) and ≥10% weight loss (78% vs. 69%). Significantly more patients with splenomegaly than patients without splenomegaly reported night sweats (65% vs. 50%), left subcostal abdominal pain or discomfort (85% vs. 37%), generalized abdominal pain (67% vs. 50%), and early satiety (79% vs. 47%) (*P* < 0.05). Bruising and shortness of breath, although less common, were identified in 39% and 30% of patients with splenomegaly, and 45% of patients (for each symptom) without splenomegaly. A high prevalence of symptoms at the time of MF diagnosis was observed across IPSS risk groups, although the overall prevalence was greater in patients with higher IPSS risk even for some symptoms that are not part of the IPSS risk stratification (Fig. 2).

Altogether, 95% of all patients had at least two symptoms of interest (Table 5). Moreover, 88% of patients with splenomegaly and 69% of patients without splenomegaly had at least four symptoms of interest, demonstrating the multisymptomatic nature of MF.

**Table 5:** Prevalence of at least one MF-related symptom at baseline

Presence of MF symptoms (*n*, %)	With splenomegaly (*n*=102)	Without splenomegaly (*n*=78)
≥1 symptom	101 (99)	77 (99)
≥2 symptoms	97 (95)	74 (95)
≥3 symptoms	95 (93)	70 (90)
≥4 symptoms	90 (88)	54 (69)

MF, myelofibrosis.

### Anemia and thrombocytopenia

Table 6 depicts the relationship between splenomegaly and anemia at baseline (or time of splenomegaly detection for patients with splenomegaly). More than 90% of patients had hemoglobin values of less than 12 g/dL, but less than 15% had severe anemia (as defined by hemoglobin levels <8 g/dL). There was no difference in the prevalence of severe anemia at baseline (9% of patients with splenomegaly vs. 14% of patients without splenomegaly). A baseline platelet count of less than 100 × 10^9^/L was noted in 69% of patients with splenomegaly versus 55% of patients without splenomegaly. Importantly, severe thrombocytopenia (platelet count of <20 × 10^9^/L) was quite rare in both cohorts (5% in patients with splenomegaly vs. 4% in patients without).

**Table 6:** Relationship between splenomegaly and baseline^1^ hemoglobin values and platelet counts in MF patients

Laboratory result	With splenomegaly (*n*=102)	Without splenomegaly (*n*=78)
Hemoglobin		
<8g/dL	9 (9)	11 (14)
8g/dL but <10g/dL	45 (44)	26 (33)
10g/dL but <12g/dL	40 (39)	34 (44)
≥12g/dL	8 (8)	7 (9)
Platelet count		
<20×10^9^/L	5 (5)	3 (4)
20×10^9^/L but <50×10^9^/L	11 (11)	10 (13)
50×10^9^/L but <75×10^9^/L	24 (24)	15 (19)
75×10^9^/L but <100×10^9^/L	30 (29)	15 (19)
100×10^9^/L but <150×10^9^/L	19 (19)	23 (29)
≥150×10^9^/L	11 (11)	12 (15)
Do not know	2 (2)	0 (0)

MF, myelofibrosis.

1 Results reported at the time of splenomegaly detection for patients with splenomegaly and at the time of MF diagnosis for patients without splenomegaly. For all test results, data could be recorded ±30days from diagnosis of splenomegaly or diagnosis of MF.

In the 12 months following MF diagnosis, 29% of patients with splenomegaly received packed red blood cell transfusion support, compared with 12% of patients without splenomegaly (*P* = 0.009). Data on hemoglobin levels at the time of each transfusion were not captured in this study.

### Pharmacologic treatment

Table 7 displays the type of pharmacotherapies administered to the study patients. Just over half of patients (52%) received pharmacologic treatment at any time after MF diagnosis and during the period of observed follow-up (44% of patients without splenomegaly; 59% of patients with splenomegaly). Those who received treatment typically initiated the medication soon after initial diagnosis (median: 39 days). The drug most commonly prescribed was hydroxyurea (59% of those on medication), followed by hematopoietic growth factors (35%), thalidomide (20%), interferon-α (16%), anagrelide (11%), and lenalidomide (6%).

**Table 7:** Medications received for MF management (at any time during the observation period)

Medication (*n*, % of total)	With splenomegaly (*n*=102)	Without splenomegaly (*n*=78)
No pharmacotherapy	42 (41)	44 (56)
Any pharmacotherapy	60 (59)	34 (44)
Hydroxyurea	36 (35)	19 (24)
Growth factors	23 (23)	10 (13)
Thalidomide	17 (17)	2 (3)
Lenalidomide	2 (2)	4 (5)
Interferon-α	12 (12)	3 (4)
Anagrelide	8 (8)	2 (3)

MF, myelofibrosis.

## Discussion

This retrospective study demonstrates a high prevalence of MF-related symptoms, such as generalized fatigue, weight loss, night sweats and fever, regardless of the presence or absence of splenomegaly. Bone pain, muscle pain, pruritus, and symptoms such as bruising and shortness of breath, although less common, also were identified in more than 30% of patients. Not unexpectedly, generalized abdominal discomfort, left subcostal pain, and early satiety were more common in patients with splenomegaly than those without splenomegaly [1]. We found that more than two-thirds of all patients had multiple symptoms, adding to the understanding of symptomatic disease burden of MF patients. Moreover, symptoms were prevalent even in patients with lower risk MF (by IPSS), underscoring the importance of comprehensive symptom evaluation in all patients with MF and limitations of the IPSS. To our knowledge, this is the first study to assess clinical features, symptoms, and treatments associated with MF in routine clinical practice, using information recorded by the physicians caring for these patients.

Although many recent reports have focused on the diagnosis [2, 17–19] and prognosis [6, 20–22] of MF patients, few published studies have systematically described the symptomatic burden among such patients. Mesa and colleagues [5] conducted an international, internet-based survey of 1179 patients with MPNs, using validated data collection instruments. Median age of the 456 responding MF patients was 58 years; 53% were female. MF patients participated in the survey a median of 6 years after diagnosis and 56% reported having splenomegaly. Symptoms reported (and percentages of patients) were fatigue (84%), night sweats (56%), itching (50%), bone pain (47%), undesired weight loss (20%), fever (18%), and spleen pain (7%). Results were not described separately for patients with and without splenomegaly. Although an internet survey could overestimate the prevalence of symptoms in a chronic disease because of patients' self-selection, our results were generally similar to those of Mesa and colleagues.

In another study, Scherber et al. [23] enrolled 128 MF patients internationally and evaluated their symptoms using the European Organization for Research and Treatment of Cancer's Quality of Life Questionnaire C30 and a new instrument, the MPN-SAF. Patients' mean age was 65 years; 46% were women. MF was diagnosed an average of 5.5 years before study assessment. Symptoms included fatigue (99% of patients), abdominal discomfort (73%), early satiety (73%), night sweats (64%), itching (51%), weight loss (48%), and fever (25%). The proportion of patients with splenomegaly was not reported, and results were not reported separately for patients with and without splenomegaly. A subsequent study of 96 patients with MF [16] evaluated the MPN-SAF in three countries: U.S., Italy, and Sweden. The proportion with splenomegaly was not reported. Symptoms included fatigue (98.9% of patients), early satiety (75.5%), abdominal discomfort (71.7%), night sweats (63.4%), bone pain (55.3%), itching (53.8%), weight loss (48.9%), and fever (29%). These investigators found that physicians' clinical assessments of patient symptoms (blinded to patients' study-collected, self-reported symptoms) correlated with their patients' responses on the MPN-SAF across all MPN types, with Pearson coefficients ranging from 0.48 for bone pain to 0.62 for itching (*P* < 0.001 for all items). In our study, some symptoms (e.g., generalized fatigue, night sweats) had similar reporting frequency as those in the Scherber et al. [16] study, whereas others were reported more often (fever, weight loss) or less often (pruritus, bone pain).

Additional information on treatment of disease-related symptomatic burden has been reported from MF clinical trials. Verstovsek and colleagues [24] reported the results of a phase 1/2 study evaluating ruxolitinib in 153 MF patients (INCB18424-251; http://ClinicalTrials.gov Identifier: NCT00509899). Patients' median age at study entry was 65 years; 37% were female. Patients were assessed a median of 6.0 years after diagnosis. As a consequence of the trial selection criteria, 92% had splenomegaly. Treatment-induced reductions in spleen size were associated with decreases in abdominal discomfort and pain. Pruritus, present in 45% of patients completing the symptom assessment questionnaire at entry, also decreased during treatment. The COMFORT-I study (NCT00952289), in which all patients were required to have splenomegaly at baseline, showed that 41.9% of patients treated with ruxolitinib experienced a 35% or greater reduction in spleen volume at week 24 compared with 0.7% of patients taking placebo (*P* < 0.0001). At this time, 45.9% of patients in the ruxolitinib group and 5.3% on placebo had 50% or greater improvement in total symptom score (*P* < 0.0001), with a median time to response of less than 4 weeks [11]. Improvements were observed in both spleen-related symptoms (abdominal pain, pain under left ribs, and early satiety) and cytokine-related symptoms (night sweats, itching, bone/muscle pain). In the COMFORT-II study (NCT00934544), the symptoms reported to occur most frequently (“quite a bit” or “very much”) at baseline were fatigue (54% of patients), dyspnea (30%), insomnia (30%), pain (29%), night sweats (23%), and itching (21%) [25]. In that trial, mean baseline (95% confidence interval) European Organisation for Research and Treatment of Cancer's global health status and quality of life (53.7 [50.6–56.7]; median: 50.0) and Functional Assessment of Cancer Therapy-General total scores (73.0 [70.8–75.2]) were comparable with scores from patients of similar age with other cancers [25]. The COMFORT-II study showed that 28.5% of patients treated with ruxolitinib experienced a 35% or greater reduction in spleen volume at 48 weeks, compared with 0.0% of patients in the best available therapy arm (*P* < 0.0001), and improvements were observed in disease-related symptoms and quality of life [12].

We compared the prevalence of symptoms among patients with splenomegaly at the time splenomegaly was first diagnosed with the prevalence of symptoms among patients presenting with MF who did not have splenomegaly. However, splenomegaly was already present at initial diagnosis of MF in the majority of patients with splenomegaly in our study, making the two groups reasonably comparable for symptom assessment. Nonetheless, it cannot necessarily be concluded that all differences in symptom frequency between the two groups were attributable directly to the presence of splenomegaly (although this explanation is likely for abdominal symptoms). Another reason for comparing symptoms at baseline is that the presence and specific combination of symptoms may change over time in an individual patient; also, symptoms may be affected by treatment, especially as newer, more effective therapies are more widely used in this patient population.

We collected information from a review of medical records, whereas other studies have gathered data on symptoms from internet-based patient surveys or direct patient interviews. Although validation results with the MPN-SAF [16] were published after our study was already initiated, our medical record review included most of the symptoms in the MPN-SAF – symptoms shown to be both prevalent and burdensome to patients with MF.

Our study is subject to several limitations inherent to retrospective medical record review studies. In particular, the patients selected for inclusion represented, to some extent, a convenience sample in that the records were obtained from physicians who were willing to participate in the study. The patients and their physicians therefore may not be fully representative of all MF patients and MF-treating physicians in the U.S. The data were entered directly into electronic forms by the treating physicians and therefore were potentially subject to error. Although automated data checks ensured internal consistency, responses were not independently validated against the actual patients' medical records. However, physicians were asked to provide additional clarifying details if any conflicting responses were found within the data collection form during a quality check process. To enhance compliance with the data collection process, the electronic form was designed to limit physicians' time burden, so it is possible that information that was not captured could be useful in understanding the disease–symptom burden in this cohort. Finally, physicians reported data from information available in the patients' charts, but patients could have received health care services in other care settings that were not reported to the treating physician (i.e., such information would have not been part of these patients' medical records) and consequently would not have been captured in this study.

This study also has important strengths. We assessed symptoms separately for patients with and without splenomegaly, allowing us to contrast the two groups – in previous studies of MF-related symptoms, these groups have been routinely combined. The data for the present study were collected from preexisting medical records of patients under care in routine practice, thereby preventing bias in symptom assessment related to any research hypothesis. Finally, the patients we evaluated were similar in several respects to other MF population samples that have been evaluated, including those enrolled in the ruxolitinib clinical trials.

The results of our study confirm a high prevalence of MF-related symptoms, many of which occur irrespective of the presence of splenomegaly or IPSS risk category. Our findings support current efforts to make available to MF patients the innovative treatments such as JAK inhibitors and other novel therapies that target both constitutional symptoms and splenomegaly-related symptoms. Future studies in larger populations are needed to provide in-depth understanding of MF manifestations and their treatments, especially as current therapy evolves for this highly symptomatic patient population.
